# Acute Gout

**Published:** 1908-02-08

**Authors:** 


### Medicine.

### ACUTE GOUT.

### AN ILLUSTRATIVE CASE.

Acute gout, with abundant deposits of urate of soda in the subcutaneous tissues, is becoming sufficiently rare to make the following typical case of some interest. The patient further exemplifies two additional points, namely: first the severity with which the stomach may be affected in gouty persons; secondly, the similar severity with which the bronchi and bronchioles may be attacked.

The patient was a man aged 43, a carter by trade. He did not appear to have been at all an intemperate man in his habits, but he had been accustomed to take his daily allowance of beer, and it is not improbable that at times he indulged fairly freely in that liquid.

The man's first attack of gout occurred as long as nine years before, when he was only 34 years of age; and it was followed by a second six months afterwards. Since that time he had not been free from an attack of gout for three months together. These attacks were, however, slight at first, and did not cause him to lie up for more than two or three days each time; possibly they were treated by colchicum; but the treatment did not prevent the frequent recurrence of the attacks, nor did it alter a character of the disease which was conspicuous in this patient from the first, namely, a tendency to shift from one joint to another. During the latter five years the paroxysms had been much longer in duration than previously, generally lasting as long as five or six weeks.

Four years before the patient came under observation, that is to say, five years from the first attack of gout, small tophi began to develop beneath the skin of the ears, and deposits of sodium urate appeared under the skin about the knuckles of each hand. These increased in size and number with each subsequent paroxysm.

When he first came under observation at hospital there was a very large collection of the "chalky" deposit on the back of the right hand; it was opened, and a great quantity of white semi-liquid matter escaped, looking like wet plaster of Paris. It was urate of soda. In this attack other symptoms of great interest besides the joint pains and the chalky deposits occurred for the first time. He had, for instance, a severe attack of bronchitis upon which the ordinary remedies seemed to exercise little or no influence, and which did not yield until after gout appeared in his feet. Soon after this he suffered from excruciating pain in the gastric region and from vomiting, with great flatulent distension of the stomach and bowels.

All these symptoms, however, passed off in time, and the patient left the hospital much improved in health after a sojourn in it of seven weeks.

He remained free from any acute attack for two years, when he was re-admitted for an attack of the disease, beginning in the little finger of the right hand and the elbow of the same side. These parts were very red and much swollen, and extremely painful. As in the previous attack, he was very much troubled with a wheezing and cough, which came on at the same time as the joint trouble; the only abnormal physical signs to note in the chest were sibilant rhonchi, and prolonged expiration like that of asthma, all over both lungs. There was moderate pyrexia, the tongue was coated with a thick fur, and the pulse-rate was 108. There was also recurrence of his former acute abdominal pain, persistent vomiting, and flatulence. The gastric symptoms were of a type suggesting the gastric crises of locomotor ataxia, but the nervous system was perfectly normal; moreover there was no blue line upon the gums nor any other sign of plumbism. Had it not been for the actual gout that was present at the same time, and for the knowledge that such attacks affecting the stomach are recognised parts of some cases of acute gout, it would have been very difficult to make a diagnosis, and exploratory laparotomy might even have been in question.

The gouty inflammation spread quickly to the other fingers, and to the whole hand, and an abscess formed in the little finger from which, when it was opened, a large quantity of pus mixed with urate of soda escaped.

The sickness increased so that everything was vomited almost as soon as it was taken. Under the use of small quantities of stimulants, and of ammonia in effervescence, these symptoms subsided; but the fingers in turn ulcerated and burst, and urate of soda was discharged in considerable abundance.

A week later, however, the abdominal pain and vomiting returned; the left hand was attacked with fresh severity by gout, the right continuing much inflamed and discharging, and there was a fresh attack of lung trouble, with dyspnœa, and a very general rhonchus over the whole chest. Soon afterwards both feet and knees became affected with gout, and copious effusions were formed in the knee joints.

Four days after this exacerbation set in, the hands and arms presented a most formidable appearance. Both hands were enlarged to nearly double their natural size, and the skin over them was red and tense; a deposit of urate of soda had formed over every joint, and in several places there were small sinuses discharging freely both pus and sodium urate. The swelling extended over the forearms, and a considerable collection of matter formed near the inner condyle of the right arm.

For several days abscess after abscess formed, each containing pus and urate of soda. These were opened in succession; and while this was going on it was found necessary to administer stimulants freely; these given in small doses at short but regular intervals were the only means found of relieving the intenseness of his gastric symptoms.

After all the abscesses had been freely opened and the urate of soda discharged in large quantities the fingers became much reduced in size, and the general constitutional disturbance quickly subsided. The rhonchi in the lungs disappeared, the vomiting ceased, the appetite returned, and the patient was enabled to eat a little solid food. The joints all improved, and the man was soon able to walk about the ward. The condition of his fingers had been extraordinarily bad before he was admitted; by the time he was discharged they were very greatly improved; for, although they were still stiff and deformed, they could now be placed close together as the result of the urate of soda deposits having been removed; and they were very much more serviceable than they had been previously.

Some of the points in connection with the above case will be more fully discussed in another article.

